# Influence of Experimental Parameters of a Continuous Flow Process on the Properties of Very Small Iron Oxide Nanoparticles (VSION) Designed for T_1_-Weighted Magnetic Resonance Imaging (MRI)

**DOI:** 10.3390/nano10040757

**Published:** 2020-04-15

**Authors:** Thomas Vangijzegem, Dimitri Stanicki, Adriano Panepinto, Vlad Socoliuc, Ladislau Vekas, Robert N. Muller, Sophie Laurent

**Affiliations:** 1 Department of General, Organic and Biomedical Chemistry, NMR and Molecular Imaging Laboratory, University of Mons, B-7000 Mons, Belgium; dimitri.stanicki@umons.ac.be; 2 Chimie des Interactions Plasma-Surface (ChIPS), University of Mons, 23 Place du Parc, B-7000 Mons, Belgium; Adriano.panepinto@umons.ac.be; 3 Laboratory of Magnetic Fluids, Center for Fundamental and Advanced Technical Research, Romanian Academy—Timisoara Branch, 300223 Timisoara, Romania; vsocoliuc@gmail.com (V.S.); vekas.ladislau@gmail.com (L.V.); 4 Research Center for Complex Fluids Systems Engineering, Politehnica University of Timisoara, M. Viteazu Ave. #1, 300222 Timisoara, Romania; 5 Center for Microscopy and Molecular Imaging, Rue Adrienne Bolland, 8, B-6041 Gosselies, Belgium; robert.muller@umons.ac.be

**Keywords:** iron oxide nanoparticles, flow synthesis, contrast agents, magnetic resonance imaging

## Abstract

This study reports the development of a continuous flow process enabling the synthesis of very small iron oxide nanoparticles (VSION) intended for T_1_-weighted magnetic resonance imaging (MRI). The influence of parameters, such as the concentration/nature of surfactants, temperature, pressure and the residence time on the thermal decomposition of iron(III) acetylacetonate in organic media was evaluated. As observed by transmission electron microscopy (TEM), the diameter of the resulting nanoparticle remains constant when modifying the residence time. However, significant differences were observed in the magnetic and relaxometric studies. This continuous flow experimental setup allowed the production of VSION with high flow rates (up to 2 mL·min^−1^), demonstrating the efficacy of such process compared to conventional batch procedure for the scale-up production of VSION.

## 1. Introduction

Due to their remarkable superparamagnetic properties and their relatively harmless nature, iron oxide nanoparticles have been at the center of several research in the biomedical field for various applications including magnetic resonance imaging (MRI) [1,2], drug delivery [3,4,5], induced magnetic hyperthermia [6,7] and cell labeling [8,9]. MRI constitutes the main application field for such material, where their use as contrast agents drastically enhances the sensitivity of the technique. These agents are generally divided in two categories depending on whether they can induce a positive (bright contrast) or a negative contrast (dark contrast) [10]. Previously used commercial contrast agents, based on superparamagnetic iron oxide contrast agents (Endorem, Resovist) [11,12], belong to the second class of contrast agents (the so-called T_2_ agents). However, the clinical uses of such agents have been limited due to disadvantages stemming from both, the nature of the magnetic nanoparticles (inducing dark contrast and prone to magnetic susceptibility artefact) [13] and the competitiveness of gadolinated contrast agent acting as T_1_ agents, which are preferred for the ease of diagnosis in clinical trials. On the other hand, concerns about the potential toxicity of gadolinium-based contrast agents have been pointed out for several years with the well-known nephrogenic systemic fibrosis (NSF) [14,15], and more recently, with cases of brain gadolinium deposition in rats [16,17]. For these reasons, the development of nanoparticulate T_1_ contrast agents, based on iron oxide nanoparticles, has become a forward-looking area of research for MRI. On the contrary to the classical clinically used T_2_ contrast agents (Endorem), very small (<5 nm) iron oxide nanoparticles (VSION) are potential candidates for T_1_-weighted imaging, due to their low magnetization and the presence of a surface spin-canted layer [18,19,20]. However, one must stress that control over the size and size distribution is an essential prerequisite to observe VSION-induced contrast enhancement on T_1_-weighted images.

In recent years, researchers have developed a wide range of synthetic procedures enabling the synthesis of superparamagnetic particles with fine control over their physicochemical properties (shape, size and size distribution) [21]. Among the existing synthesis protocols, the thermal decomposition of organometallic precursors (iron complex) has emerged, over time, as the most efficient method for the preparation of highly calibrated iron oxide nanoparticles. The typical thermal decomposition method relies on the decomposition of an iron complex (iron oleate, iron acetylacetonate) in high boiling point organic solvent (i.e., dibenzylether, oleylalcohol) in the presence of organic surfactants (i.e., oleylamine, oleic acid). Although the method has proven its efficacy, the high level of control required for synthesis, and the harsh experimental conditions, could be limiting factors, especially for the exploitation of the process on larger scales [22].

Bearing in mind these issues, researchers have focused on developing new alternatives, in order to avoid these limitations. For some years, we have witnessed the emergence of continuous flow processes for the preparation of inorganic nanoparticles [23]. Due to a very high surface to volume ratio, flow reactors provide substantial advantages over the conventional batch procedures, very rapid heat transfer enables fast cooling or heating of a solution, as well as precise temperature control [24]. These features are particularly interesting for the implementation of the thermal decomposition synthesis of iron oxide nanoparticles.

In this study, we developed a continuous flow system preparation of VSION by thermal decomposition. A parametric study is proposed, focusing on the influence of different experimental parameters (concentration and nature of surfactants, temperature, pressure, residence time, and capillary inner diameter) on nanoparticles characteristics, such as the size, the magnetic and relaxometric properties by means of the study of their nuclear magnetic resonance dispersion (NMRD) profiles.

## 2. Experimental Section

### 2.1. Materials

Oleylamine (98%), oleic acid (90%), dibenzylether (99%), iron(III) acetylacetonate (99.9%), nitric acid and hydrogen peroxide were purchased from Sigma-Aldrich (Overijse, Belgium). Oleyl alcohol (>60%) and 1,2-hexadecanediol (98%) were purchased from TCI Chemicals (Zwijndrecht, Belgium). Ethanol (EtOH; 100%) and tetrahydrofuran (THF; 99.9%) were purchased from Chemlab (Zedelgem, Belgium). All the materials mentioned above were used without further purification.

### 2.2. Batch Synthesis of VSION

Typically, a mixture of oleic acid (2 mmol; 635 μL), oleylamine (2 mmol; 658 μL) and 1,2-hexadecanediol (10 mmol; 2.58 g) was solubilized in oleyl alcohol (10 mL) and heated at 300 °C for 10 min under nitrogen and magnetic stirring. Then, a solution of iron (III) acetylacetonate (2 mmol; 706 mg) in oleyl alcohol (10 mL) was rapidly injected into the flask. The mixture was heated at 300 °C for a further 30 min, then rapidly cooled. The particles were isolated after pouring an excess of ethanol (40 mL) into the cooled solution, followed by magnetic decantation. Finally, the as-obtained precipitate was redispersed in tetrahydrofuran (10 mL) and centrifuged to remove any undissolved materials (16.000 g; 10 min).

### 2.3. Flow Synthesis of VSION

A flow-adapted thermal decomposition process was used for the synthesis of very small iron oxide nanoparticles. In typical experiment, the following stock solution was prepared: iron acetylacetonate (10 mmol; 3.53 g), oleylamine (40 mmol; 10.7 g; 13.2 mL) and oleic acid (40 mmol; 11.3 g; 12.6 mL) were mixed in a 1:1 mixture of dibenzylether and oleyalcohol to reach a final volume of 100 mL (iron precursor concentration of 100 mM). This stock solution was preheated at 70 °C to form a homogenous solution. The stock solution was then pumped using a High Performance Liquid Chromatography (HPLC) pump through a 1 m polytetrafluoroethylene (PTFE) tube reactor immerged in a heating device where the temperature was maintained at elevated temperatures (temperatures of 200, 225, 250, 275 and 300 °C). The system pressure was controlled by a back-pressure regulator assembly (BPRA). Five BPRA inducing counter-pressure values of 5, 20, 40, 75 and 100 psi were evaluated. The effect of surfactant concentration and surfactant nature was studied by varying the composition of the stock solution. The total surfactant concentration was varied between 40 and 120 mM using three different conditions: (i) Oleic acid alone, (ii) oleylamine and oleic acid in equimolar concentration, and (iii) Oleylamine alone. The effect of residence time/flow rate and capillary internal diameter were investigated using tubing with internal diameters (ID) of 0.5, 0.75, 1 and 2.4 mm and flow rates of 0.05, 0.1, 0.5, and 1 mL/min and 2 mL/min* (* Only for the 2.4 mm (ID) PTFE reactor). After being cooled to room temperature, the particles were isolated by pouring an excess of a 1:1 mixture of ethanol and acetone (40 mL) into the suspension, followed by magnetic decantation. Finally, the as-obtained precipitate was resuspended in tetrahydrofuran and centrifuged to remove any undissolved materials (16.000 g; 10 min).

### 2.4. Characterizations

Transmission electron microscopy (TEM) was used to obtain detailed morphological information and was carried out using a Microscope Fei Tecnai 10 operating at an accelerating voltage of 80 kV (Oregon, OR, USA). The samples were prepared by placing a drop of diluted suspension on carbon-coated copper-grid (300 mesh; NP in organic media), allowing the liquid to dry in air at room temperature. The statistical treatment of the TEM images was performed by iTEM (Münster, Germany) on multiple images for each sample. The mean diameter, the standard deviation and the polydispersity index (PDI) were calculated by measuring the particle diameter. The number of nanoparticles counted ranged from 300 to 500.

The measurements of the size distribution of the nanoparticles suspended in organic medium were performed on a Zetasizer nano zs (Malvern Instruments, Malvern, UK) using laser He-Ne (633 nm). For each measurement, the suspensions were diluted in THF to approximately 1 mM in iron concentration.

Nuclear Magnetic Relaxation Dispersion (NMRD) profiles were recorded on samples in organic media with a field cycling relaxometer (STELAR, Mede, Italy) measuring the longitudinal relaxation rates (R_1_) in a magnetic field range extending from 0.24 mT to 1 T. The temperature of the samples was adjusted to 37 °C with a precision of 0.1 °C. The theoretical adjustment of the NMRD profiles was performed using classical relaxation models [25,26] assuming a diffusion coefficient of THF of 3.27 × 10^−9^ cm^2^·s^−1^ [27].

Longitudinal (R_1_) and transverse (R_2_) relaxation rate measurements at 0.47 and 1.41 T were obtained on Minispec mq 20 and mq 60 spin analysers (Bruker, Mannheim, Germany) respectively. The relaxation rates were measured as a function of the iron molar concentration at 0.47 and 1.41 T, in order to calculate the r_1_ and r_2_ relaxivities (defined as the enhancement of the relaxation rate of water protons in 1 mmol·L^−1^ solution of contrast agents). The relaxivities were calculated as the slope of relaxation rate (R_i_ obs) versus iron concentration according to the equation,

(1) $$R_{i}^{obs}=\frac{1}{T_{i}^{obs}\;}=r_{i}\left[Fe\right]+\frac{1}{T_{i}^{dia}}$$

with *r*_i_ being the relaxivities and $T_{i}^{dia}$ being the proton relaxation times of the solvent without nanoparticle.

The total iron concentration was determined by the measurement of the longitudinal relaxation rate R_1_ according to the method previously described [28]. Briefly, the samples were mineralized by microwave digestion (MLS-1200 Mega, Milestone, Analis, Belgium) and the R_1_ value of the resulting solutions was recorded at 0.47 T and 37 °C, which determined the iron concentration using equation,

[Fe] = (R_1_^sample^ − R_1_^dia^) × 0.0915 (2)

where R_1_^dia^ (s^−1^) is the diamagnetic relaxation rate of acidified water (0.36 s^−1^) and 0.0915 (s·mM) is the slope of the calibration curve.

The yield was determined after measuring the iron content of purified suspensions. The yield is defined as:

(3) $$\mathrm{Reaction}\;\mathrm{yield}=\frac{n_{Fe}}{n_{Fe\;Stock\;solution}}\times 100\%$$

where *n*_Fe_ is iron molar content in the purified suspension, *n*_Fe Stock solution_ is the iron molar content in the stock solution.

The magnetization of the samples was measured by means of Vibrating Sample Magnetometry (VSM) at room temperature using an ADE Technologies VSM880 magnetometer (Massachusetts, MA, USA). The samples saturation magnetization was determined as the intercept of the linear fit of M = M(1/H) at H large (i.e., 1/H → 0) [29]. The magnetic diameter distribution of the nanoparticles was obtained by means of magnetogranulometry, i.e., the nonlinear regression of the magnetization curve with Ivanov and coworkers second order modified mean field theory for highly concentrated polydisperse samples [30]. The log-normal distribution was assumed for nanoparticles magnetic diameter [31],

(4) $$f\left(D\right)=\frac{1}{D\sigma \sqrt{2\pi }}\cdot e^{-\frac{1}{2\sigma^{2}}\cdot {\left(ln\frac{D}{D_{0}}\right)}^{2}}$$

where *D*_0_ and σ are the fit parameters. Using *D*_0_ and *σ*, the calculation of the mean diameter (<*D*>) and standard deviation (StDev) is straight forward.

The phase constitution of the powders was evaluated by X-ray diffraction (XRD) using a PANalytical Empyrean diffractometer working with Cu K_α1_ radiation (*λ* = 0.1546 nm) at an incidence angle, Ω, of 3°. The powders were continuously rotated during the measurements to ensure a homogeneous exposure to the X-Rays. The resulting patterns were compared to the JCPDS card of magnetite Fe_3_O_4_ (JCPDS card, No. -080-6407). The X-ray source voltage was fixed at 45 kV and the current at 40 mA. The grain size (*G*_s_) was calculated from the most intense diffraction peak, i.e., [311] using the following Scherrer equation [32],

(5) $$G_{s}=\frac{K\;ʎ}{\beta \;cos\;\theta }$$

where *K* is a dimensionless shape factor, *ʎ* is the X-ray wavelength, *β* is the line broadening at half the maximum (FWHM) and *θ* is the Bragg angle.

## 3. Results and Discussion

### 3.1. VSION Formation by Thermal Decomposition Using Batch Process

**Figure 1:**
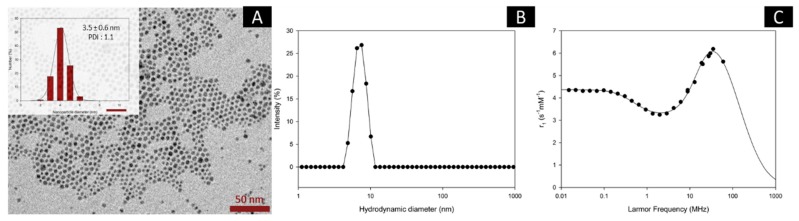
TEM image of the VSION in THF. The scale bar corresponds to 50 nm. The inset shows the size distribution: 3.5 ± 0.6 nm (PDI: 1.1) (**A**); Size distribution in intensity as measured by PCS (**B**); ^1^H NMRD profile, recorded at 37 °C in THF (**C**).

In standard batch conditions, two experimental procedures are commonly used for the thermal decomposition process: the “heating-up” [33] and the “hot-injection” methods [34]. Both procedures allow the formation of nearly monodisperse iron oxide nanoparticles, with tunable sizes, by simply varying the experimental parameters based on the precise separation between the nucleation and the growth steps involved in the formation mechanism of the nanocrystals [35]. For the preparation of VSION, we decided to apply a previously established process [20] which consisted in the direct injection of a solution containing the iron organometallic complex solubilized in oleylalcohol in a solution of hydrophobic surfactants (e.g., oleic acid, oleylamine and 1,2-hexadecanediol in a 1:1:5 molar ratio) in a dibenzylether solution preheated at 300 °C. As a result, VSION with well-defined properties were readily obtained. Figure 1A presents a TEM image of the resulting nanoparticles which show spherical morphology and exhibiting an average diameter of 3.5 ± 0.6 nm (PDI: 1.1). The monodispersity of the sample was also confirmed by photon correlation spectroscopy (PCS), for which a narrow size distribution has been observed (Figure 1B), as well as a hydrodynamic diameter centered at around 7 nm (PDI: 0.090).

The relaxation properties of magnetic nanoparticles were obtained by the study of their nuclear magnetic resonance dispersion profiles (NMRD profiles; Figure 1C). These curves show the evolution of the proton longitudinal relaxation (r_1_) as a function of the applied magnetic field (Larmor frequency). The shape of this profile corresponds to the typical curve expected for superparamagnetic colloidal suspensions. At low field, the small dispersion is a main feature of small-sized individual iron oxide nanoparticles characterized by a low anisotropy energy [25]. After this dispersion, the hump present at stronger magnetic field is due to the evolution of the Curie magnetization. The fit of the NMRD curve using the classical relaxation models allowed to estimate a saturation magnetization (MSAT of 39.6 A·m²·kg^−1^) and a NMRD diameter (D_NMRD_ of 6.24 nm). One could underline the good correlation between D_NMRD_ and D_PCS_. Relaxometric properties of batch-prepared VSION, Dotarem® and Resovist® are shown in Table S1.

### 3.2. Influence of Experimental Parameters on Nanoparticles Formation by Thermal Decomposition Using Flow Process

**Figure 2:**
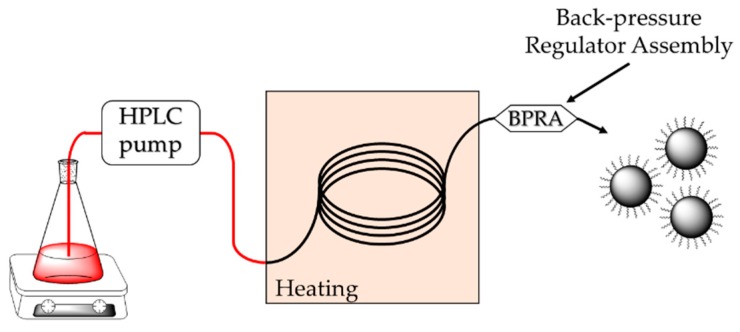
Schematic representation of the experimental setup used for the flow synthesis of VSION.

The flow synthesis was performed using PTFE tube reactors with a fixed length (1 m) and internal diameters of 0.5, 0.75, 1, and 2.4 mm. Figure 2 depicts the experimental setup designed for the flow synthesis of the nanoparticles. It consists of a HPLC pump operating at flow rates between 10 µL·min^−1^ and 10 mL·min^−1^ which inject the reaction mixture in a PTFE tubing heated at constant temperature. The system pressure is controlled by a back-pressure regulator assembly (BPRA) located at the output of the capillary. The as-synthesized nanoparticles were cooled at room temperature at the exit of the capillary and then collected. Any unreacted iron complexes, as well as surfactant excess were removed by means of several washing steps using ethanol/acetone mixture. After redispersion in THF, the as-obtained suspensions were finally centrifuged to remove any undissolved materials.

Due to the high surface-to-volume ratios characteristic of flow reactors [22], fast heat transfer can be achieved in order to emulate the hot-injection batch process. This sudden increase of temperature induces the decomposition of the iron organometallic precursor into iron monomers (iron oxo-clusters) [36,37] leading to a burst nucleation phenomenon. Subsequent growth of these nuclei results in the formation of iron oxide nanoparticles in the capillary.

In order to adapt and optimize the synthesis to the continuous flow process, slight modifications were made to the batch recipe. The major one implied the withdrawing of 1,2-hexadecanediol owing to its low solubility in the reaction mixture at room temperature (to avoid clogging of the HPLC pump). As a result, some adjustments in the oleic acid/oleylamine ratio had to be done.

### 3.3. Influence of the Ligand/Precursor Molar Ratio and Ligand Nature

Among the numerous studies treating about the thermal decomposition for the synthesis of iron oxide nanoparticles, many reports show the strong impact of the surfactant/precursor molar ratio on the properties of the obtained nanoparticles with different tendencies reported by several research groups [38,39]. In order to evaluate the influence of the surfactant concentration and the surfactant nature, the flow synthesis has been conducted with different ratios of (i) oleic acid only, (ii) oleic acid and oleylamine and (iii) oleylamine only. All the experiments were carried out in the same solvent mixture (i.e., dibenzylether and oleylalcohol 1/1) and three surfactants/precursor molar ratios were tested (i.e. 4, 8 and 12 equivalents of surfactants). Experiments were carried out using a temperature of 250 °C and BPRA of 40 psi.

Iron oxide nanoparticles synthesized with the three different concentrations of pure oleic acid exhibited very poor stability in THF, leading to diluted suspensions with very low yields (less than 10%). Moreover, the TEM images (and the corresponding histograms) (Figure S1; SI) show nanoparticles with no well-defined shape and very broad size distributions (PDI > 1.3). These results correlate well with other report [40] in which the synthesis of iron oxide nanoparticles using oleic acid as surfactant resulted in low yields. The lack of stabilisation using oleic acid was recently investigated by Harris et al. [41] using molecular mechanics modelling to evidence lowest binding energy between oleic acid and the nanoparticle’s surface compared to systems combining oleic acid and oleylamine. In their study, they demonstrated that oleic acid only cannot yield to stable suspensions. It was suggested the binding of deprotonated oleic acid molecules onto the nanoparticles leads to a neutral average surface charge and at the same time to an increase in free proton concentration in the media. As a consequence of these two phenomena, the zeta potential will overtime tend toward zero and the formed nanoparticles tend to agglomerate.

**Figure 3:**
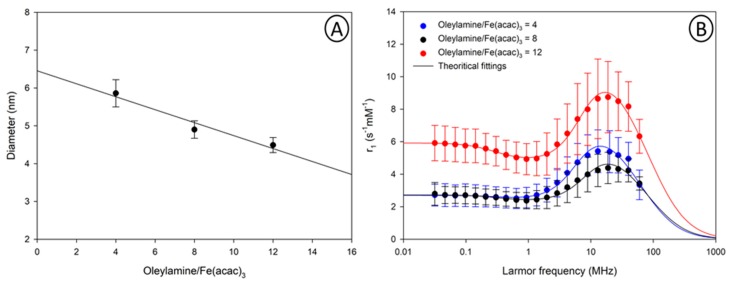
(**A**)Evolution of TEM diameter as a function of the oleylamine amount; (**B**)^1^H NMRD profiles of samples obtained by flow synthesis with various amount of oleylamine.

Interestingly, when oleylamine was used as surfactant. A linear decrease of the mean particle size when increasing oleylamine amount was observed (Figure 3A). Qi et al. [42] reported a similar trend using three different aliphatic amines (hexylamine, oleylamine and octadecylamine). However, substantial differences are observed in the NMRD profiles recorded for these samples (Figure 3B). Indeed, smaller nanoparticles exhibit higher longitudinal relaxivity on the overall range of Larmor frequency.

Although these results seem contradictory with the superparamagnetic relaxation theory, differences in the magneto-crystalline state of the nanoparticles could explain this phenomenon. Oleylamine, can act as a reducing agent besides its role as a capping agent. The presence of a large excess of oleylamine can therefore provide a strong reductive environment affecting the crystallinity of the obtained nanoparticles [43]. The magnetic properties of nanoparticles were evaluated, using VSM measurements, and compared to the parameters extracted from the fitting of the NMRD profiles (Table S3; SI). Again, a good correlation between the M_S_ values, measured by VSM and the M_S_ values, determined from the superparamagnetic theory, was obtained. Therefore, these results demonstrate that tuning the oleylamine to iron precursor ratio is an experimental parameter, which can be easily implemented to produce iron oxide nanoparticles with variable sizes and variable degrees of crystallinity, using our continuous flow process.

**Figure 4:**
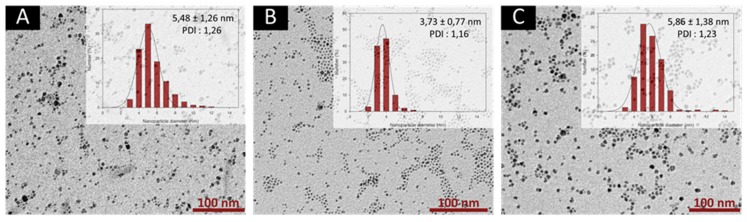
TEM images of the nanoparticles obtained through flow synthesis with various equivalents of oleic acid and oleylamine: Two equivalents (**A**), four equivalents (**B**) and six equivalents (**C**) in the 1 mm capillary reactor. The scale bar corresponds to 100 nm. Insets show the size distributions determined by statistical analysis.

On the other hand, conducting the synthesis with various amounts of oleylamine and oleic acid (in equimolar quantities), yielded to stable and concentrated colloidal suspensions. Size changes were measured with TEM (Figure 4). These results show that a ratio of surfactants to iron precursor of 4:1 leads to smaller nanoparticles with narrower size distribution compared to synthesis performed with larger (6:1) and lesser (2:1) amounts of surfactants. This suggests that there is an optimum ratio at which the organic surfactants provide a better stability to the nuclei formed in situ, and is therefore, favourable to the formation of smaller nanoparticles having appropriate relaxometric properties (r_2_/r_1_ ratios) (Table S2; SI). Therefore, the surfactants to iron precursor of 4:1 was used for the study of the other experimental parameters, described in the following sections.

### 3.4. Influence of Temperature

**Figure 5:**
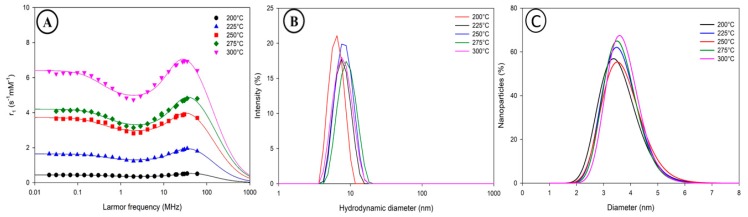
^1^H NMRD profiles of iron oxide nanoparticles obtained at various temperatures recorded at 37 °C in THF (**A**). The continuous lines correspond to the fitting by the superparamagnetic relaxation theory; Size distribution in intensity as measured by PCS of VSION obtained at various temperatures (**B**); size distributions as determined by TEM (**C**).

To study the influence of the temperature, synthesis experiments were carried out using a PTFE tube reactor with an internal diameter of 1 mm and at a flow rate of 1 mL·min^−1^. In a typical synthesis, a BPRA inducing a back pressure of 40 psi was used. Synthesis were carried out using oleic acid and oleylamine as surfactants (with a surfactant to precursor ratio of 4/1) considering the results abovementioned (i.e., flow synthesis using four equivalents of oleic acid and oleylamine in equimolar concentration). Five batches were prepared by working at temperatures of 200, 225, 250, 275 and 300 °C. Surprisingly, no significant differences in the mean diameter has been observed neither by PCS nor by TEM. The resulting suspensions were all characterized by mean hydrodynamic diameters below 10 nm (Figure 5B; Table S4; SI) and a mean inorganic diameter below 4 nm (Figure 5C) accompanied with polydispersity indexes between 1.10 and 1.16.The relaxometric properties of these samples were investigated by the study of their NMRD profiles (Figure 5A). 

The results indicate a marked increase of the relaxivity values on the overall range of Larmor frequency when increasing the temperature in the capillary reactor. The samples obtained at lower temperature (i.e., 200 and 225 °C) exhibit very low values of longitudinal relaxivities. Despite the significant differences observed in their relaxometric properties, the small dispersion observed for each sample still indicates that their anisotropy energy remains low and characteristic of VSION. Theoretical parameters (M_SAT_ and D_NMRD_) extracted from the fitting of these profiles are shown in Table S4 along with the nanoparticle sizes determined by PCS and TEM. The estimated D_NMRD_ values are in good correlation with the hydrodynamic sizes measured by PCS. Taken together, these theoretical and experimental measurements indicate no influence of the temperature on the size of the synthesized nanoparticles. However, the extracted values of M_SAT_ are markedly increasing with the reaction temperature (Table S4; SI). At this stage, the formulated hypothesis relies on differences in the magneto-crystalline state of the VSION. Previous report from Belaid et al. [44] showed the same tendency when synthesizing iron oxide nanoparticles in classical thermal decomposition synthesis at different temperatures. XRD and VSM measurements showed the increase of the particles crystallinity degree with the temperature, giving rise to increased relaxometric properties.

Since the iron oxide nanoparticles obtained at a temperature of 250 °C display relaxometric properties similar to those of obtained for batch samples, this temperature was chosen as the working temperature for the following flow syntheses. Moreover, this temperature was chosen due to the nature of the capillary reactor i.e., PTFE which is known to be thermally resistant up to 250 °C (for long-term continuous service) [45].

### 3.5. Influence of Pressure

**Figure 6:**
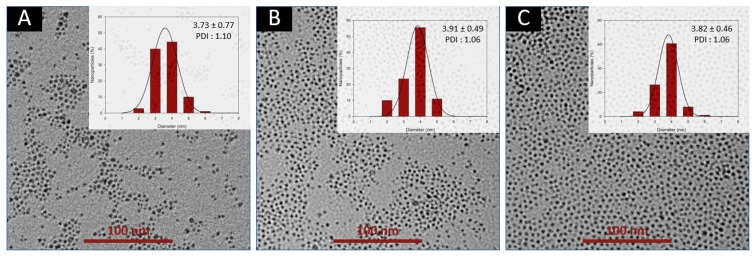
TEM images of the nanoparticles obtained through flow synthesis with BPR of 40 psi (**A**), 75 psi (**B**) and 100 psi (**C**). The scale bar corresponds to 100 nm. Insets show the size distributions determined by statistical analysis.

The influence of pressure on the particles properties was studied using back-pressure regulator assemblies inducing counter-pressure varying between 5 and 100 psi (BPR of 5, 20, 40, 75 and 100 psi were tested). With the 5 and 20 psi BPR, flow cavitation was observed in the capillary reactor and the resulting nanoparticles were characterized by very low relaxometric properties (Table 1). On the other hand, BPR of 40, 75 and 100 psi delivered good flow stability and a steady flow was straightforwardly reached in the capillary reactor. The TEM images of VSION obtained with these three BPR are presented in Figure 6. Size histograms were fitted using a log-normal function and standard deviation and polydispersity index were calculated.

For each sample, VSION with a mean diameter smaller than 4 nm are observed. NMRD profiles of these three samples of VSION (Figure S2; SI) are stackable, showing no incidence of the pressure on both, the size and relaxometric properties of the obtained VSION (Table S5). Therefore, we concluded that pressure only influences the stability of the flow in the capillary reactor and we investigated the influence of other parameters such as flow rate and residence with the 40 psi BPR.

### 3.6. Influence of Residence Time in the Capillary Reactor

**Figure 7:**
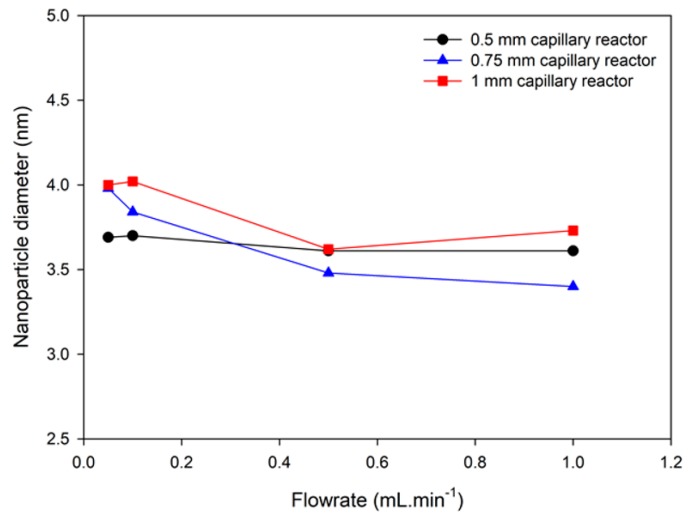
Average diameter of VSION as a function of flowrate in capillary reactors with different internal diameters.

The residence time in the capillary reactor was modified by varying two experimental parameters, i.e., the capillary inner diameter and the flow rate. The influence of these flow parameters was initially investigated by using three capillary reactors with internal diameters of 0.5; 0.75 and 1 mm. For each capillary, experiments have been conducted with four flow rates (0.05; 0.1; 0.5 and 1 mL·min^−1^). Photon correlation spectroscopy analysis confirmed for each sample the formation of nanoparticles characterized by a narrow monomodal distribution with a mean diameter below 10 nm. Determination of nanoparticles size and polydispersity index was performed using TEM (Figure 7).

**Figure 8:**
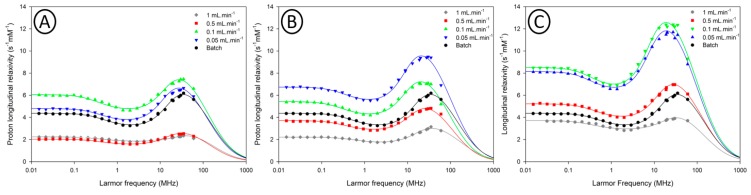
^1^H NMRD profiles of VSION obtained in the 0.5 mm capillary reactor (**A**), the 0.75 mm capillary reactor (**B**) and 1 mm capillary reactor (**C**). The black curve is the NMRD profile of the batch-prepared VSION. The continuous lines correspond to the fitting by the superparamagnetic relaxation theory.

No matter the experimental conditions, it was observed that the nanoparticle diameter remained roughly constant when working at different flow rates. All samples are characterized by mean diameter between 3.4 and 4 nm and no significant tendency of size increase is observed when decreasing the flow rate. Such observations are quite different from other report from Jiao et al. [46], whereby a significant impact of the residence time on the size properties of iron oxide nanoparticles was reported using a similar flow process. This suggests a different mechanism of nanoparticle formation using our flow process. The decomposition of the iron precursor depends on the solvents and the amounts of surfactants present in the media. In our experimental setup, the reaction conditions seem to lead to a burst nucleation phenomenon yielding VSION with constant size. However, if no clear influence was observed by TEM, significant differences were observed when analysing the NMRD profiles (Figure 8).

Within each capillary reactor, we observe a global increase of the relaxometric properties of the VSION when using slower flow rate. The increase of the residence time induces an increase of proton longitudinal relaxivity in the overall studied range of Larmor frequency. Notably, is the shift of the hump (maximum proton longitudinal relaxivity) towards lower frequency values when slower flow rates are used, this indicates that an increased residence time is favourable to slightly larger nanoparticles, which is not observed from the TEM analysis. This could also be provoked by a structural change with different heating time. This observation can be confirmed by the NMRD diameters extracted by the theoretical fittings of the NMRD profiles (Table 1). The NMRD diameters are much higher than the sizes determined by TEM, given that the superparamagnetic relaxation theory was developed for crystalline superparamagnetic compounds, the hypothesis explaining this observation would rely on differences in the crystalline state of the nanoparticles. Nanoparticles synthesized at higher flow rates have a residence time of less than 2 min in the capillary reactor. Such short residence time might not be sufficient to yield well-crystallized iron oxide nanoparticles. These differences in the nanoparticles relaxometric properties can, therefore, be attributed to differences in the nanoparticle’s crystallinity degree and consequently to their magnetic and relaxometric properties. Nonetheless, when compared to their batch-prepared derivatives, VSION prepared at slower flow rates exhibit similar or even better relaxometric properties (high longitudinal relaxivity values and low r_2_/r_1_ ratios) for applications as T_1_ contrast agents (Table 2).

**Figure 9:**
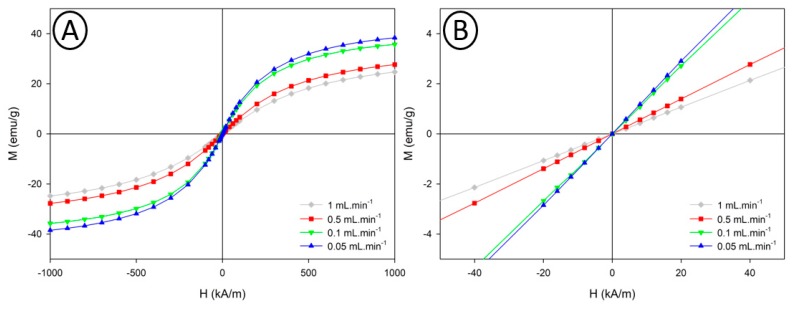
Magnetization curves of samples obtained in the 1 mm capillary reactor as a function of the applied magnetic field (**A**); Zoomed region around origin (**B**).

To confirm these differences in the magnetic properties of the iron oxide nanoparticles, magnetometry measurements were conducted on samples obtained in the 1 mm capillary reactor. Those results are presented in Figure 9.

Magnetization curves obtained for VSION synthesized at all flow rates show the superparamagnetic nature of the nanoparticles for each sample. As demonstrated in Figure 9, all samples have a superparamagnetic behavior characterized by the absence of hysteresis (absence of coercivity and absence of remanent magnetization). Fitting the magnetometry data using Ivanov 2nd order modified field theory [30] allowed the the magnetic diameter distribution to be determined. Saturation magnetization (MS) was measured using Chantrell method [29] (Table S6; SI). The results are well fitted with the relaxometry measurements (M_S_ and D_NMRD_ obtained by the fitting of the NMRD profiles). From these results, a same trend towards the increase of the magnetic properties when working at slower flow rates was observed.

**Figure 10:**
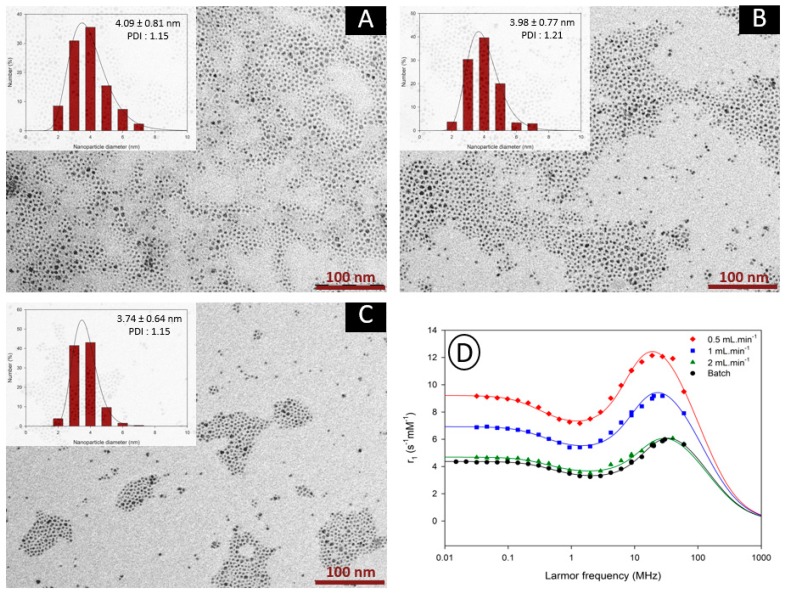
TEM images of the nanoparticles obtained through flow synthesis with flow rates of 0.5 mL·min^−1^ (**A**), 1 mL·min^−1^ (**B**) and 2 mL·min^−1^ (**C**) in the 2.4 mm capillary reactor. The scale bar corresponds to 100 nm. Insets show the size distributions determined by statistical analysis; (**D**) ^1^H NMRD profiles of the three samples (colored curves) and the batch-prepared sample (black curve). The continuous lines correspond to the fitting by the superparamagnetic relaxation theory.

To go further in the parametric study and the production objective intended for the synthesis of VSION, the influence of flow parameters was investigated using a capillary with a larger internal diameter (2.4 mm). Considering the dead volume of this capillary, flow rates of 0.5, 1 and 2 mL·min^−1^ were used. TEM images (and corresponding histograms fitted using a log-normal function) of samples obtained at flow rates of respectively 0.5, 1 and 2 mL·min^−1^ are presented in Figure 10 with the corresponding NMRD profiles recorded for each sample.

TEM and relaxometry results lead to the same conclusions: flow rate and consequently the residence time do not have a marked influence on the size properties of the as-synthesized nanoparticles. For all three samples, nanoparticles were characterized by a mean size around 4 nm and relatively low polydispersity index. However, these three samples exhibit interesting relaxometric properties compared to their batch-prepared derivatives. As seen on the NMRD profiles, samples obtained at 0.5 mL·min^−1^ and 1 mL·min^−1^ exhibit proton longitudinal relaxivity higher than the batch sample over the whole range of proton Larmor frequencies. In addition, sample obtained at 2 mL·min^−1^ and the batch sample display analogous values of longitudinal relaxivities, confirming that a residence time of approximately 2 min is sufficient to yield VSION with properties similar to batch-prepared ones.

Furthermore, by comparing the properties of all samples obtained in tubings with different internal diameters, no clear proportionality between the residence time and the nanoparticles properties can be pointed out. Indeed, even if a global increase of the relaxometric properties with the residence time is observed for each capillary. Samples obtained with similar residence time in capillaries with different internal diameters do not exhibit properties (Table 2). An additional parameter which could potentially explain these discrepancies concern the thickness of the PTFE tubings. All tubings are made of 1/8 PTFE with varying internal diameters. As a result, the wall thickness is not the same for each tubing. This thickness difference might affect the heat transfer in the tubing and therefore affect the properties of the synthesized nanoparticles.

**Figure 11:**
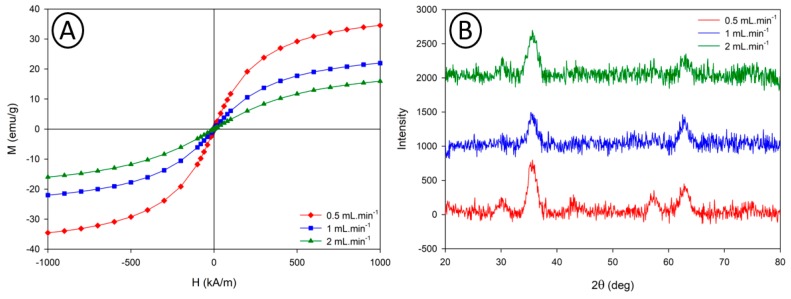
Magnetization curves (**A**) and XRD diffraction patterns (**B**) of samples obtained in the 2.4 mm capillary reactor.

As mentioned above, these differences in relaxometric properties may be attributed to differences in the nanoparticle’s magneto-crystalline properties. To confirm these differences in the crystalline state potentially affecting the magnetic and therefore the relaxometric properties of the VSION, VSM measurements were performed on the three samples, but also X-ray diffraction experiments, to obtain a qualitative estimation of the crystallinity of the three samples (Figure 11).

Similarly to the VSION synthesized in the 1 mm PTFE capillary, the VSION are all characterized by a superparamagnetic behaviour and the magnetization saturations obtained by the Chantrell method follow the same trend as previously shown with the 1 mm capillary reactor. Differences in the crystallinity state of these 3 samples was confirmed using X-ray diffraction experiments. The characteristics diffraction peaks observed at 30.2°, 35.5°, 42.8°, 57.2° and 62.7° and their relative intensities match well with the standard XRD data of magnetite Fe_3_O_4_ (JCPDS card No. 080-6407). The XRD peak intensity increases with the residence time in the capillary reactor, combined with the line broadening for samples obtained at higher flow rate (indicating the presence of smaller crystallites when using shorter residence time), this demonstrates the presence of nanoparticles with increased crystallinity when working at slower flow rate. An estimation of the magnetite nanoparticles size was made with both, characterization techniques, using magnetogranulometry and the Scherrer formula (taking the highest intensity peak corresponding to the [311] plane at 2*θ* = 35.5°) for VSM, and XRD, respectively (Table 3).

Whereas, the estimated size of the nanoparticles, determined by magnetogranulometry, is in relatively good agreement with the TEM diameter, the estimated particle diameters measured from the XRD measurements, using the Scherrer formula are slightly higher than the measured size determined from the TEM analysis, this overestimation of the nanoparticles size might be due to the very small size of the nanoparticles limiting the use of the Scherrer equation.

## 4. Conclusions

We have developed a continuous flow process for the thermal decomposition synthesis of very small iron oxide nanoparticles. The influence of experimental parameters (ligand concentration and nature, temperature, pressure capillary inner diameter and residence time) was investigated to determine their inflluence on the nanoparticles magneto-crystalline and relaxometric properties. This study showed that experimental parameters have almost no incidence on the nanoparticles size properties. However, a clear impact was evidenced based on the relaxometric and magnetic properties of the particles. As a consequence, nanoparticles obtained with increased residence times exhibit higher relaxometric and magnetic properties. With respect to the application of these VSION for MRI, an important feature defining their efficacy as T_1_ contrast agents is their r_2_/r_1_ ratios. The ideal T_1_ MRI contrast agent is characterized by high r_1_ values and low r_2_/r_1_ ratios in order to reach their highest imaging efficacy [47,48]. In our case, VSION synthesized using this flow process exhibit higher r_1_ values than commercially used T_1_ contrast agents [49] such as Dotarem® or other paramagnetic agents as well as r_2_/r_1_ ratios below three at standard clinical fields (1.5 T). This flow process allowed the production of VSION with superior throughput than classical batch methods.

## Figures and Tables

**Table 1:** Relaxometric properties of VSION obtained at different pressures.

BPRA (psi)	20 MHz		60 MHz	
	r_1_ (s^−1^mM^−1)^	r_2_ (s^−1^mM^−1)^	r_1_ (s^−1^mM^−1)^	r_2_ (s^−1^mM^−1)^
5	0.5	0.7	0.6	1.2
20	1.4	1.9	1.5	3.3
40	3.7	5.3	3.7	8.8
75	3.6	5	3.7	8.6
100	3.6	5	3.7	8.6

**Table 2:** Summary of the experimental parameters and results obtained by PCS, TEM and relaxometry for the study of flow parameters influences.

Capillary Inner Diameter (mm)	Flow Rate(mL·min^−1^)	Residence Time (min)	Diameter			NMRD Data		Relaxivity Measurements	
			D_PCS_ (nm)	D_TEM_ (nm)	PDI	D_NMRD_ (nm)	M_s_ (A·m²·kg^−1^)	r_2_/r_1_ (20 MHz)	r_2_/r_1_ (60 MHz)
0.5	0.05	3.93	6.1	3.69 ± 0.81	1.16	6.82	38.4	1.58	2.42
0.5	0.1	1.96	6.2	3.70 ± 0.84	1.14	6.50	41.2	1.54	2.30
0.5	0.5	0.39	8.8	3.70 ± 0.92	1.15	7.18	25.4	1.45	2.26
0.5	1	0.20	6.6	3.61 ± 0.72	1.15	5.82	27	1.40	2.19
0.75	0.05	8.84	9.1	3.98 ± 1.00	1.23	7.34	41.6	1.57	2.63
0.75	0.1	4.42	8.8	3.84 ± 0.76	1.16	7.76	34.8	1.56	2.60
0.75	0.5	0.88	8.1	3.48 ± 0.76	1.20	7.28	31	1.56	2.53
0.75	1	0.44	6.8	3.40 ± 0.82	1.23	5.68	30.5	1.50	2.49
1	0.05	15.71	7.1	4.00 ± 0.94	1.19	7.52	44.8	1.63	2.55
1	0.1	7.85	9.5	4.02 ± 0.80	1.14	7.52	46.6	1.73	2.74
1	0.5	1.57	8.1	3.62 ± 0.74	1.17	6.70	39.8	1.58	2.48
1	1	0.79	7.7	3.73 ± 0.77	1.10	6.96	33.9	1.53	2.43
2.4	0.5	9	7.3	4.09 ± 0.81	1.15	7.44	47.3	1.84	2.69
2.4	1	4.5	9.3	3.98 ± 0.77	1.21	7.04	44.2	1.63	2.59
2.4	2	2.25	8.1	3.74 ± 0.64	1.15	6.62	38.6	1.51	2.46

**Table 3:** Summary of the results obtained with TEM, VSM and XRD for samples obtained in the 2.4 mm capillary reactor.

Flow Rate (mL·min^−1^)	Diameter			M_SAT_ (emu/g)
	D^TEM^ (nm)	D^VSM^ (nm)	D^XRD^ (nm)	
0.5	3.71 ± 0.76	4.16 ± 0.91	5.02	40.1
1	3.98 ± 0.77	3.90 ± 0.76	4.59	26.4
2	3.74 ± 0.64	3.51 ± 0.58	4.16	20.4

## Supplementary Materials

The following are available online at https://www.mdpi.com/2079-4991/10/4/757/s1. Table S1: Relaxometric properties of Dotarem®, Resovist® and batch-prepared VSION; Figure S1: TEM images of the nanoparticles obtained through flow synthesis with various equivalents of oleic acid: 4 equivalents (left), 8 equivalents (middle) and 12 equivalents (right) in the 1 mm capillary reactor. The scale bar corresponds to 100 nm. Insets show the size distributions determined by statistical analysis; Table S2: Size and relaxometric properties of VSION synthesized using various surfactant concentrations (oleic acid and oleylamine); Table S3: Size and magnetic properties of VSION synthesized using various oleylamine concentrations; Table S4: Size and relaxometric properties of VSION synthesized at different temperatures using continuous flow process; Table S5: size and magnetic properties of VSION synthesized using different BPR; Figure S2: ^1^H NMRD profiles of iron oxide nanoparticles obtained at various pressure recorded at 37 °C in THF; Table S6: Magnetic properties extracted from the fitting of the magnetization curves of samples obtained in the 1 mm capillary reactor.
