# Supplementary material for: Influence of Experimental Parameters of a Continuous Flow Process on the Properties of Very Small Iron Oxide Nanoparticles (VSION) Designed for T1-Weighted Magnetic Resonance Imaging (MRI)
Source: Nanomaterials (Basel). 2020 Apr 15;10(4):757. doi: 10.3390/nano10040757 (PMC7221927; doi:10.3390/nano10040757)
Supplement: Supplementary file 1 [file nanomaterials-10-00757-s001.pdf]

Supplementary Material

Influence of Experimental Parameters of a Continuous Flow Process on the Properties of Very Small Iron Oxide Nanoparticles (VSION) Designed for T_1-weighted Magnetic Resonance Imaging (MRI)

Thomas Vangijzegem [1,*], Dimitri Stanicki [1], Adriano Panepinto [2], Vlad Socoliuc [3], Ladislau Vekas [3,4], Robert N. Muller [5] and Sophie Laurent [1,5]

1 Department of General, Organic and Biomedical Chemistry, NMR and Molecular Imaging Laboratory, University of Mons, B-7000 Mons, Belgium; dimitri.stanicki@umons.ac.be (D.S.); sophie.laurent@umons.ac.be (S.L.)

2 Chimie des Interactions Plasma-Surface (ChIPS), University of Mons, 23 Place du Parc, B-7000 Mons, Belgium; Adriano.panepinto@umons.ac.be

3 Laboratory of Magnetic Fluids, Center for Fundamental and Advanced Technical Research, Romanian Academy—Timisoara Branch, 300223 Timisoara, Romania; vsocoliuc@gmail.com (V.S.); vekas.ladislau@gmail.com (L.V.)

4 Research Center for Complex Fluids Systems Engineering, Politehnica University of Timisoara, M. Viteazu Ave. #1, 300222 Timisoara, Romania

5 Center for Microscopy and Molecular Imaging, Rue Adrienne Bolland, 8, B-6041 Gosselies, Belgium; robert.muller@umons.ac.be

* Correspondence: thomas.vangijzegem@umons.ac.be; Tel.: +32-(0)65-373-525

Received: 12 March 2020; Accepted: 30 March 2020; Published: date

Table S1. Relaxometric properties of Dotarem®, Resovist® and batch-prepared VSION

Sample	20 MHz			60 MHz		
	r_1 ($s^{-1}mM^{-1}$)	r_2 ($s^{-1}mM^{-1}$)	r_2/r_1	r_1 ($s^{-1}mM^{-1}$)	r_2 ($s^{-1}mM^{-1}$)	r_2/r_1
VSION	5.6	8.3	1.48	5.7	12.8	2.24
Dotarem®	3.7	4.4	1.19	3.1	3.6	1.16
Resovist®	24.9	176.8	7.10	10.9	190.2	17.45

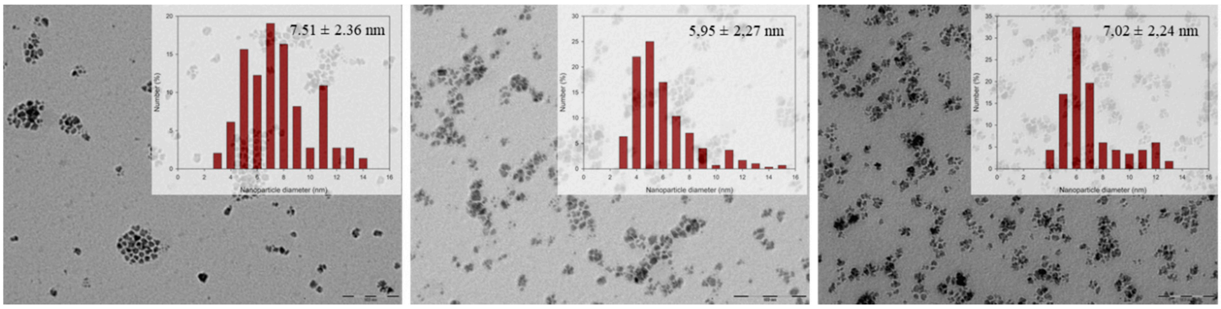

Figure S1. TEM images of the nanoparticles obtained through flow synthesis with various equivalents of oleic acid: 4 equivalents (left), 8 equivalents (middle) and 12 equivalents (right) in the 1 mm capillary

reactor. The scale bar corresponds to 100 nm. Insets show the size distributions determined by statistical analysis

Table S2. Size and relaxometric properties of VSION synthesized using various surfactant concentrations (oleic acid and oleylamine)

Oleic acid/Oleylamine/$Fe(acac)_3$	D_{PCS} (nm)	D_{TEM} (nm)	PDI	r_2/r_1 (20 MHz)	r_2/r_1 (60 MHz)
2/2/1	10.7	5,48 ± 1,26	1.26	1.76	3.03
4/4/1	7.7	3,73 ± 0,77	1.16	1.53	2.43
6/6/1	10.4	5,86 ± 1,38	1.23	1.67	3.1

Table S3. Size and magnetic properties of VSION synthesized using various oleylamine concentrations

Oleylamine/$Fe(acac)_3$	M_{SAT} ($A{\cdot}m^2/kg$)	$\boldsymbol{M_{SAT}^{NMRD}}$ ($A{\cdot}m^2/kg$)	D_{NMRD} (nm)
4/1	29.4	24.9	9.98
8/1	28.1	25.5	8.78
12/1	40.1	35.9	8.34

Table S4. size and relaxometric properties of VSION synthesized at different temperatures using continuous flow process

Temperature (°C)	D_{PCS} (nm)	M_{SAT} ($A{\cdot}m^2/kg$)	D_{NMRD} (nm)	D_{TEM} (nm)
200	6.5	11.7	7.2	3.54 ± 0.76
225	7.9	22	6.8	3.68 ± 0.60
250	7.7	33.9	6.2	3.73 ± 0.77
275	9.2	37.7	6	3.53 ± 0.54
300	8	41.7	6.3	3.74 ± 0.55

Table S5. size and magnetic properties of VSION synthesized using different BPR

BPR	D_{PCS} (nm)	M_{SAT} ($A{\cdot}m^2/kg$)	D_{NMRD} (nm)	D_{TEM} (nm)
5 psi	8.6	---	---	---
20 psi	6.7	---	---	---
40 psi	7.7	33.9	6.2	3.73 ± 0.77
75 psi	7.7	32.3	6.3	3.91 ± 0.49
100 psi	8.9	32.3	6.3	3.82 ± 0.46

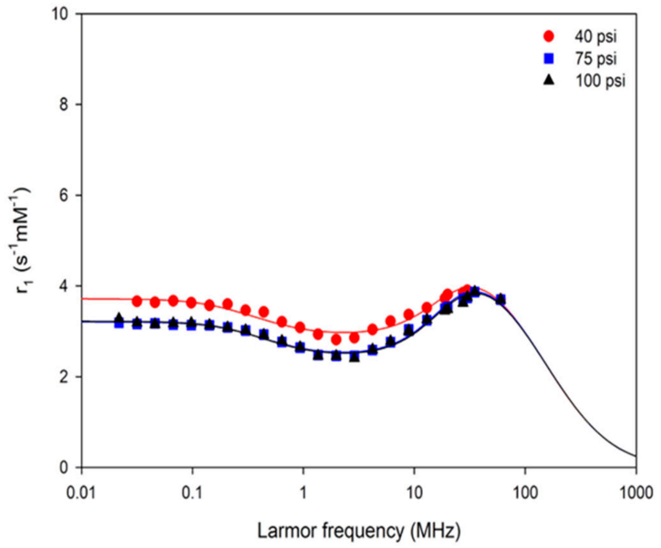

Figure S2. ^{1}H NMRD profiles of iron oxide nanoparticles obtained at various pressure recorded at 37 °C in THF.

Table S6. Magnetic properties extracted from the fitting of the magnetization curves of samples obtained in the 1 mm capillary reactor.

**Flow rate (mL·min^{-1})**	**Magnetometry data**	
	D (nm)	M_S (emu·g^{-1})
0.05	3.89 ± 0.94	45.2
0.1	3.94 ± 0.95	41.9
0.5	3.61 ± 0.72	34.5
1	3.46 ± 0.4	31.6
